# Development of a Rapid Salivary Proteomic Platform for Oral Feeding Readiness in the Preterm Newborn

**DOI:** 10.3389/fped.2017.00268

**Published:** 2017-12-12

**Authors:** Prarthana Khanna, Jill L. Maron, David R. Walt

**Affiliations:** ^1^Sackler School of Graduate Biomedical Sciences, Tufts University School of Medicine, Boston, MA, United States; ^2^Mother Infant Research Institute, Floating Hospital for Children at Tufts Medical Center, Boston, MA, United States; ^3^Department of Pathology, Brigham and Women’s Hospital, Wyss Institute for Biologically Inspired Engineering, Harvard Medical School, Boston, MA, United States

**Keywords:** protein, biomarker, saliva, adenosine-monophosphate-activated protein kinase, neuropeptide Y2 receptor

## Abstract

Oral feeding competency is a major determinant of length of stay in the neonatal intensive care unit. An infant must be able to consistently demonstrate the ability to take all required enteral nutrition by mouth before discharge home. Most infants born prematurely (<37 weeks) will require days, if not weeks, to master this oral feeding competency skill. Inappropriately timed feeding attempts can lead to acute and long-term morbidities, prolonged hospitalizations, and increased health-care costs. Previously, a panel of five genes involved in essential developmental pathways including sensory integration (*nephronophthisis 4, Plexin A1*), hunger signaling [*neuropeptide Y2 receptor* (*NPY2R*), *adenosine-monophosphate-activated protein kinase* (*AMPK*)], and facial development (*wingless-type MMTV integration site family, member 3*) required for oral feeding success were identified in neonatal saliva. This study aimed to translate these five transcriptomic biomarkers into a rapid proteomic platform to provide objective, real-time assessment of oral feeding skills, to better inform care, and to improve neonatal outcomes. Total protein was extracted from saliva of 10 feeding-successful and 10 feeding-unsuccessful infants matched for age, sex, and post-conceptional age. Development of immunoassays was attempted for five oral feeding biomarkers and two reference biomarkers (GAPDH and YWHAZ) to normalize for starting protein concentrations. Normalized protein concentrations were correlated to both feeding status at time of sample collection and previously described gene expression profiles. Only the reference proteins and those involved in hunger signaling were detected in neonatal saliva at measurable levels. Expression patterns for NPY2R and AMPK correlated with the gene expression patterns previously seen between successful and unsuccessful feeders and predicted feeding outcome. Salivary proteins associated with hunger signaling are readily quantifiable in neonatal saliva and may be utilized to assess oral feeding readiness in the newborn. This study lays the foundation for the development of an informative, rapid, proteomic platform to assess neonatal oral feeding maturation.

## Introduction

Although oral feeding competency is a discharge requirement from the neonatal intensive care unit (NICU), there is currently a paucity of objective assessment tools to determine oral feeding maturity in this population (1, 2). Rather, standard of care is largely limited to subjective assessment of an infant’s feeding cues (i.e., ability to suck on a pacifier) once an infant corrects to >32 weeks’ post-conceptional age (PCA) and has a stable respiratory status (3–6). The absence of an objective assessment tool to determine oral feeding readiness has not only placed immature oral feeders at risk for significant feeding associated morbidities, including choking, poor growth, impaired short- and long-term neurodevelopmental outcomes, and feeding aversions, but it has also resulted in prolonged hospitalization, increased health-care costs, and parental anxiety (7–11).

Previously, salivary gene expression analyses on hundreds of premature infants at both pre- and post-oral feeding success, identified five genes involved in oral feeding maturity that were differentially expressed between successful and unsuccessful oral feeders (12). These genes included *Plexin A1* (*PLXNA1*), *neuropeptide Y2 receptor* (*NPY2R*), *adenosine-monophosphate-activated protein kinase* (*AMPK*), *wingless-type MMTV integration site family, member 3* (*WNT3*), and *nephronophthisis 4* (*NPHP4*). While this prior study demonstrated the feasibility of utilizing saliva as a non-invasive biofluid to detect transcriptomic biomarkers associated with neonatal developmental milestones, the ability to monitor these markers in a timely manner to inform care remains a challenge. Proteins have numerous benefits over mRNA transcripts including their relative abundance and stability (13). Combined with their relative ease for detection and quantification compared to genes, proteins are more appealing for the development of a rapid salivary diagnostic platform. This study aimed to translate the previously described gene expression panel to a salivary proteomic platform to rapidly and objectively assess oral feeding readiness to limit neonatal morbidities and improve outcomes.

## Materials and Methods

### Infant Recruitment and Saliva Collection

This study was approved by the Tufts Medical Center Institutional Review Board. Parents of premature infants ranging from 33 to 39 weeks’ PCA were informed about the study and provided written consent before enrollment. The Tufts Medical Center NICU utilizes the cue-based feeding assessment protocol of Ludwig and Waitzman (14). In accordance to this protocol, infants with a stable respiratory status and PCA of >32 weeks were assessed for feeding capability as part of their routine vital signs by the nursing staff. This cue-based feeding assessment protocol scores infants from 1 to 5 (1 signifies oral feeding ready and 5 signifies no oral feeding cues present). Infants who demonstrate a score ranging from 1 to 2 consecutively for three assessment points are allowed to attempt oral feeds. No infant less than 32 weeks’ PCA was offered oral feeding in the NICU, thus, saliva was not collected until the infant was mature enough to attempt oral feeds (e.g., >32 weeks’ PCA). Saliva samples were collected equally from successful (*n* = 10) and unsuccessful (*n* = 10) oral feeders at a single time-point. Unsuccessful oral feeders, termed “non-feeders,” took <50% of feeds by mouth; successful oral feeders, termed “feeders,” took full (100%) oral feeds. The <50% of feeds by mouth cutoff was utilized to ensure that extraneous factors (e.g., nursing staff ratios or acute medical emergencies that may have prohibited an oral feeding session for the infant) did not contribute to an infant’s designated feeding status. Only those infants who consistently demonstrated an oral intake of <50% of full enteral nutrition were deemed unsuccessful oral feeders. The infants from both cohorts were matched for sex, gestational age (GA), PCA at time of sample collection, and ethnicity to limit the potential confounding effects of these variables on protein expression (Table 1).

**Table 1:** Patient demographics.

Feeder	Sex	Post-conceptional age (PCA)	Gestational age (GA)	Non-feeder	Sex	PCA	GA
1	F	39 2/7	37 0/7	1	F	38 5/7	38 2/7
2	F	33 4/7	32 1/7	2	F	32 6/7	32 2/7
3	F	37 2/7	34 0/7	3	F	37 0/7	36 2/7
4	F	34 4/7	33 4/7	4	F	34 2/7	33 1/7
5	F	35 1/7	32 5/7	5	F	35 2/7	33 5/7
6	M	35 2/7	31 5/7	6	M	34 6/7	34 1/7
7	M	37 2/7	36 6/7	7	M	34 1/7	33 0/7
8	M	35 0/7	33 1/7	8	M	34 3/7	32 6/7
9	M	39 2/7	37 4/7	9	M	39 1/7	37 6/7
10	M	35 3/7	34 5/7	10	M	34 6/7	33 0/7

Two saliva samples were collected for protein analysis from a single time-point in all subjects. Salivary protein was collected, stabilized, and extracted from each sample using established protocols (15). Final elution volume following extraction was 250 μl, making it necessary to collect two samples from each infant to have sufficient volumes for downstream experiments (required volume: 120 μl per biomarker). Total protein extracted from the salivary samples was stored at −80°C pending analysis.

### Development of Rapid Proteomic Platform

Immunoassays were used to measure target protein molecules in a sample. These assays were based on the specific recognition of target molecules by both capture and detection antibodies. For detection of proteins, the sandwich format was used due to its high specificity and ability to analyze biofluids in complex matrices (16). For protein immunoassays, antibody pairs and recombinant protein standards were utilized. In brief, capture antibodies were immobilized on microspheres that could be suspended in solution. The microspheres were incubated for 40 min with the sample to allow for protein-specific antibody binding. Subsequently, detection antibody was added to the solution that then bound to another epitope on the target protein. The microspheres were washed and then incubated for 20 min with 2.5 μg/ml of a streptavidin−phycoerythrin conjugate (Columbia Biosciences) to generate the fluorescent complex. After a final wash and resuspension in 75 μl phosphate buffer saline tween-20, the assay results were evaluated on the Tecan Infinite M200 Plate Reader platform. Serial dilutions of recombinant protein standards for all biomarkers were run to generate calibration curves and to determine assay sensitivity, antibody performance, and protein detection range. In addition, salivary sample dilution series and spike-in and recovery experiments were run for all developed assays to exclude non-specific binding and to determine the effect of the saliva matrix on the proteins. Manufacturer’s details and catalog numbers for all antibodies and recombinant protein standards used are provided in Table S1 in Supplementary Material.

### Testing of Rapid Proteomic Platform

Sandwich assays were performed for the detection of protein biomarkers in neonatal saliva as described earlier. Alongside the recombinant protein standards’ assays, the microspheres were also incubated with neonatal saliva samples obtained from feeders and non-feeders. The fluorescence intensity for all samples was measured on the Tecan Infinite M200 Plate Reader, and their protein concentrations were determined based on the calibration curves on the same plates.

### Data Analysis

GraphPad Prism was used to generate calibration curves for all biomarkers. These curves were fit using a 4PL fit with 1/y^2^ weighting factor. The calibration curves were used to determine concentrations of all protein biomarkers from the neonatal saliva samples. GAPDH and YWHAZ were used as reference proteins to normalize varying neonatal salivary protein concentrations and serve as quality control indices. Detection of both reference proteins was required for a sample to be considered in our analysis (17–19). Samples were normalized for comparative analysis with the use of the geometric mean (GM) of the two reference protein concentrations, using the following formula (example shown below is for AMPK):

ΔAMPK(n)=AMPK(n)/GM [GAPDH(n)+YWHAZ(n)].

## Results

**Figure 1:**
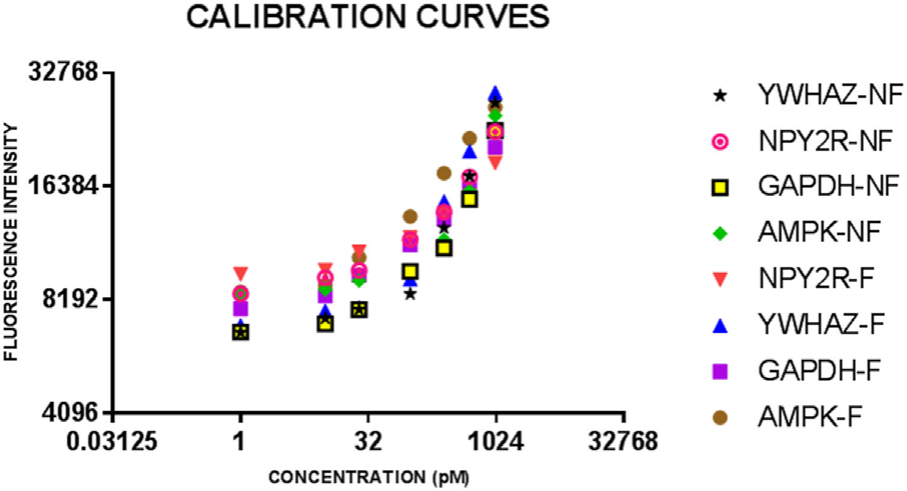
Calibration curves for eight bulk assays for salivary neonatal biomarkers. Serial dilutions of recombinant protein standards for adenosine-monophosphate-activated protein kinase (AMPK) and neuropeptide Y2 receptor (NPY2R) were run on all plates for feeder and non-feeder infant samples. Error bars depict the SDs for the values as they were all run in triplicate.

**Figure 2:**
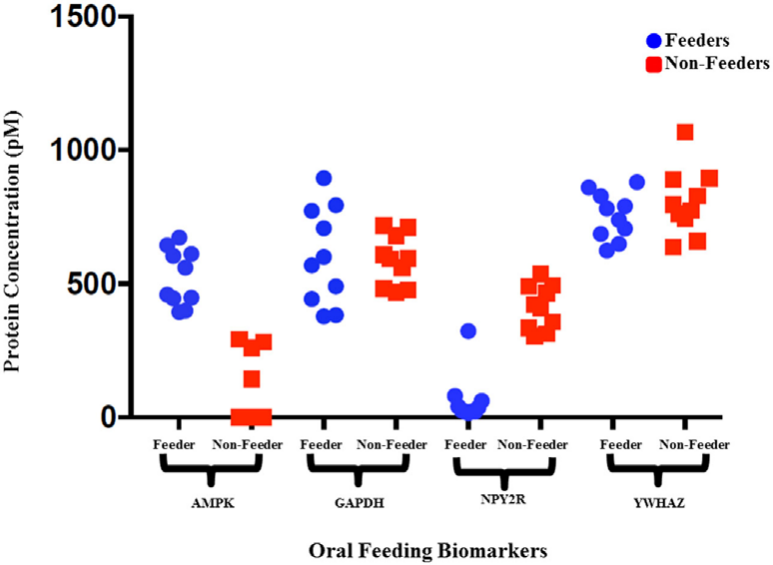
Raw protein expression levels for salivary neonatal biomarkers. Saliva samples from 10 feeders and 10 non-feeders were run using bulk immunoassays for adenosine-monophosphate-activated protein kinase (AMPK), GAPDH, neuropeptide Y2 receptor (NPY2R), and YWHAZ. Salivary protein concentrations (pM) were calculated using the calibration curves and plotted. The wide variation observed in protein expression of reference biomarkers GAPDH and YWHAZ signify varying protein input between samples.

**Figure 3:**
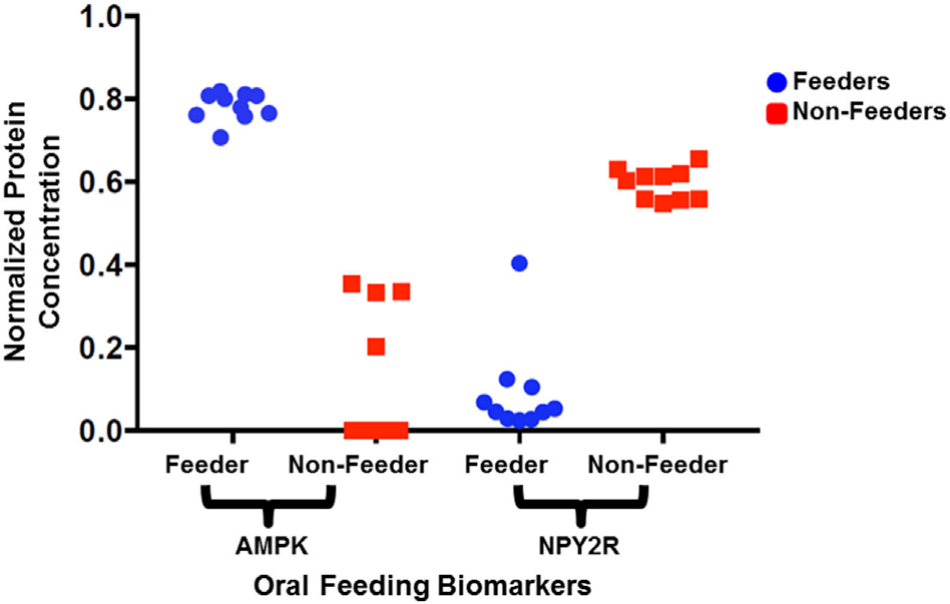
Normalized protein expression levels for salivary neonatal biomarkers. The protein concentrations present in the clinical samples for adenosine-monophosphate-activated protein kinase (AMPK) and neuropeptide Y2 receptor (NPY2R) were normalized against GAPDH and YWHAZ and plotted for all 10 feeders and all 10 non-feeders.

Immunoassays were successfully developed for five of the seven biomarkers: AMPK, NPY2R, WNT3, GAPDH, and YWHAZ. No compatible antibody pairs were found for NPHP4 and PLXNA1. Assay development for all biomarkers is summarized in Table 2. Results for salivary sample dilution series and spike-in and recovery experiments are summarized in Table S2 in Supplementary Material. Calibration curves for all assays are shown in Figure 1. Raw and normalized data are depicted in Figures 2 and 3, respectively.

**Table 2:** Assay overviews.

Protein	Assay developed	Protein detected
Neuropeptide Y2 receptor	Y	Y
Adenosine-monophosphate-activated protein kinase	Y	Y
Plexin A1	N	N
Nephronophthisis 4	N	N
Wingless-type MMTV integration site family, member 3	Y	N
GAPDH	Y	Y
YWHAZ	Y	Y

All neonatal saliva samples met quality control as defined by detection of both reference proteins in a sample. Subject demographics and pertinent clinical data are summarized in Table 1. Protein levels of both AMPK and NPYR mirrored the gene expression profiles reported previously. Namely, AMPK was either undetectable (*n* = 6) or decreased (*n* = 4) in unsuccessful oral feeders, while expression levels of NYP2R were increased in unsuccessful oral feeders (*n* = 10). Median concentrations for AMPK and NPY2R in 20 neonatal saliva samples split by feeders and non-feeders are summarized in Table 3. WNT3 was undetectable in the neonatal saliva samples analyzed.

**Table 3:** Median concentrations (pM) of the two measured biomarkers in 20 neonatal saliva samples split by feeders and non-feeders.

Biomarkers	Adenosine-monophosphate-activated protein kinase (AMPK) (feeders)	AMPK (non-feeders)	Neuropeptide Y2 receptor (NPY2R) (feeders)	NPY2R (non-feeders)
Median (pM)	510	271	31.95	415
Detectable samples	10/10	4/10	10/10	10/10

## Discussion

The transcriptome and proteome, unlike the genome, provide insight into biological function and phenotype. Transcription and translation are complex mechanisms consisting of stochastic expression levels of RNA and protein over time. Numerous studies have shown that there is not a direct correlation between the levels of mRNA and protein (20–23). However, to date, no study has used transcriptomic information as a guide for determining whether or not proteins are expressed. Using information from the neonatal salivary transcriptome, we hypothesized not only that the same proteins would be present and correlate with mRNA expression levels but also that this information would advance our understanding of the dynamic relationship between the transcriptome and the proteome in the developing premature newborn.

Previously, we identified five key regulatory genes responsible for oral feeding maturity that were differentially expressed between successful and unsuccessful oral feeders, including *NPY2R, AMPK, PLXNA1, NPHP4*, and *WNT3* (12). In this prior work, each biomarker was expressed in a binary fashion (i.e., it was either present or absent as ascertained by amplification). With mRNA expression, an infant demonstrated a mature oral feeding pattern when *AMPK, PLXNA1*, and *NPHP4* were present, and when *NPY2R* and *WNT3* were absent in neonatal saliva. Using these data, we hypothesized that the protein expression for these targets would parallel their gene expression and allow for translation to a more rapid proteomic bedside assay platform to assess oral feeding readiness.

A rapid proteomic bedside platform for assessing oral feeding readiness has the potential to limit hospital length of stay and dramatically reduce health-care costs. Two specific groups of neonates, in particular, may largely benefit from such a diagnostic assay. First, utilizing this assay to identify neonates with mature oral feeding skills, in a timely fashion, who could begin oral feeding trials without the fear of deleterious side effects, would likely reduce hospital length of stay. Second, this assay could be utilized to understand the developmental pathways limiting oral feeding success in infants who struggle to orally feed despite an advancing PCA. This approach would allow caregivers to personalize care plans based specifically on an infant’s salivary profile. Here too, there is an important opportunity to expedite oral feeding maturation and reduce time spent in the NICU. With average NICU costs at $3500 per day in the US, successful development of this rapid $5 assay to assess oral feeding maturation may result in health-care cost savings of billions of dollars per year.

In this pilot study, assays were successfully developed for five of the seven biomarkers including AMPK, NPY2R, WNT3, GAPDH, and YWHAZ. We were unable to develop protein-based assays for all the biomarkers previously identified because of a lack of suitable binding reagents for all the proteins. When these five immunoassays were carried out on newborn saliva, only the two reference biomarkers (GAPDH and YWHAZ) and the two proteins involved in hunger signaling (NPY2R and AMPK) were detectable. WNT3, associated with facial development, was undetectable. Each of the two detectable proteins paralleled the gene expression results previously described. In contrast to the gene expression levels, where the biomarkers were informative in a binary fashion, proteins could be measured at levels such that we could quantify them. This discrepancy may be due to each respective assay’s detection limit or because of differences between mRNA expression levels and protein abundance in neonatal saliva. The biological significance of this finding is unknown.

Of the three proteomic biomarkers assessed in this study, only those involved in hunger signaling were readily detectable in neonatal saliva. Their presence suggests not only that they may play an important role in regulating feeding behavior in the newborn but also implies that protein levels in saliva may be required for a maturing gut–brain axis necessary for successful oral feeding. NPY2R was first described in 1996; it is an appetite hormone and candidate gene for obesity development and control of food intake (24–26). Although one feeder’s NPY2R expression (F6) remained an outlier after normalization, this result could be attributed to the subject’s earlier GA, 31 5/7, compared to the other subjects (GA: 32 1/7 to 38 2/7).

Similar to NPY2R, AMPK expression may also play a key regulatory role in feeding maturation. AMPK detects and maintains metabolic energy balance by promoting ATP production and facilitating the pathways involved in circadian rhythms of metabolism and feeding behavior (27, 28). Expression of these two protein biomarkers corresponded not only to their gene expression profiles previously reported but were predictive of the feeding status of the newborn. Thus, they have the potential to serve as independent and reliable biomarkers of neonatal oral feeding success.

Limitations of this study include the small sample size and the inability to detect all the biomarkers previously shown to be indicative of oral feeding readiness in the newborn. The assay’s ability to detect only biomarkers involved in hunger signaling hinders its current applicability at the bedside. Biomarker proteins corresponding to other key developmental milestones required for oral feeding success including neurodevelopment, gastrointestinal maturation, and sensory integration will need to be identified before the assay will reach its full diagnostic potential. Nevertheless, this study is a promising first step toward the development of a NICU bedside device to assess oral feeding maturation to improve care and outcomes in this population. Finally, it is important to note that the assay requires only a small amount of neonatal saliva, which is easy to obtain and avoids blood collection—a cause of morbidity in neonates.

In conclusion, this pilot study is not only clinically relevant because we show concordance between protein and gene expression but we also demonstrate that protein expression can be informative of oral feeding status. Thus, for these particular biomarkers, a rapid proteomic assay may be utilized to assess real-time hypothalamic feeding development using neonatal saliva.

## Ethics Statement

This study was carried out in accordance with the recommendations of the Institutional Review Board at Tufts Medical Center with written informed consent from all subjects. Parents of all subjects gave written informed consent in accordance with the Declaration of Helsinki. The protocol was approved by the Institutional Review Board at Tufts Medical Center.

## Author Contributions

PK, JM, and DW contributed extensively to the work presented in this paper. PK carried out the experiments and wrote the manuscript with support from JM and DW. JM and DW helped to supervise the project.

## Disclaimer

This work has not been published or presented previously.

## Conflict of Interest Statement

The authors declare that the research was conducted in the absence of any commercial or financial relationships that could be construed as a potential conflict of interest.
