# Supplementary material for: Development of a Rapid Salivary Proteomic Platform for Oral Feeding Readiness in the Preterm Newborn
Source: Front Pediatr. 2017 Dec 12;5:268. doi: 10.3389/fped.2017.00268 (PMC5733069; doi:10.3389/fped.2017.00268)
Supplement: Supplementary file 1 [file Table_1.DOCX]

**Supplemental Data**

Supplementary Table 1: Manufacturer’s details and catalog numbers for all antibodies and recombinant protein standards

| Target Protein | Capture Antibody | Detection Antibody | Recombinant Protein |
| --- | --- | --- | --- |
| AMPK | Fisher/R&D Systems DYC3197 | Fisher/R&D Systems DYC3197 | Fisher/R&D Systems DYC3197 |
| GAPDH | Fisher/R&D Systems DYC5718 | Fisher/R&D Systems DYC5718 | Fisher/R&D Systems DYC5718 |
| YWHAZ | Fisher/R&D Systems DY2669 | Fisher/R&D Systems DY2669 | Fisher/R&D Systems DY2669 |
| NPY2R | Sigma-Aldrich SAB2500707 | LSBio (Direct) LS-C264678 | ABCAM AB152580 |
| WNT3 | ABCAM AB52568 | Novus Biologicals, LLC H00007473-D01PB | ABCAM AB132336 |
