# Supplementary material for: Development of a Rapid Salivary Proteomic Platform for Oral Feeding Readiness in the Preterm Newborn
Source: Front Pediatr. 2017 Dec 12;5:268. doi: 10.3389/fped.2017.00268 (PMC5733069; doi:10.3389/fped.2017.00268)
Supplement: Supplementary file 2 [file Table_2.DOCX]

**Supplemental Data**

Supplementary Table 2: Assay validations

| Protein | 0pM + 250ul saliva | | 0pM + 125ul saliva | |  | 10pM | 100pM | 1000pM | 1000pM  (+ 1hr) |
| --- | --- | --- | --- | --- | --- | --- | --- | --- | --- |
|  | | **Protein Detected** | | **% Recovery** | | | | | |
| AMPK  (in neonatal saliva) | **Y** | | **N** | |  | **108.20%** | **87.70%** | **105.50%** | **100.70%** |
| GAPDH  (in neonatal saliva) | **Y** | | **N** | |  | **98.40%** | **111.30%** | **82.30%** | **89.30%** |
| NPY2R  (in neonatal saliva) | **Y** | | **N** | |  | **117.50%** | **109.40%** | **109.90%** | **100.10%** |
| YWHAZ  (in neonatal saliva) | **Y** | | **N** | |  | **102.10%** | **99.30%** | **97.00%** | **91.90%** |
